# Changes in intramuscular blood flow and muscle deoxygenation at different parts of the vastus lateralis during intermittent isometric knee extension in young adult males

**DOI:** 10.14814/phy2.70783

**Published:** 2026-03-26

**Authors:** Kazuma Izumi, Keisho Katayama, Yutaka Kano, Noriko Tanaka, Hiroshi Akima

**Affiliations:** ^1^ Graduate School of Education and Human Development Nagoya University Nagoya Aichi Japan; ^2^ Research Center of Health, Physical Fitness and Sports Nagoya University Nagoya Aichi Japan; ^3^ Graduate School of Medicine Nagoya University Nagoya Aichi Japan; ^4^ Department of Engineering Science, Bioscience and Technology Program University of Electro‐Communications Chofu Tokyo Japan

**Keywords:** intramuscular blood flow, muscle deoxygenation, near‐infrared spectroscopy, power Doppler ultrasonography, regional difference

## Abstract

Regional differences in oxygen supply–demand balance in active muscles may be related to exercise tolerance. However, the dynamics of oxygen supply–demand balance and its regional differences within active muscles have not been clarified. The purpose of this study was to evaluate regional differences in the patterns of change in the relationship between intramuscular blood flow and muscle deoxygenation along the vastus lateralis length during submaximal isometric knee extension. Thirteen healthy young males (19.4 ± 0.9 years) performed intermittent (5‐s contraction and 5‐s relaxation) isometric knee extension at 50% of maximal voluntary contraction for 18 contractions. Intramuscular blood flow and deoxy‐[hemoglobin+myoglobin] (deoxy‐[Hb+Mb]) were simultaneously measured using power Doppler ultrasonography and near‐infrared spectroscopy, respectively, at the proximal, middle and distal sites of the vastus lateralis. The ratio of intramuscular blood flow to deoxy‐[Hb+Mb] (intramuscular blood flow/deoxy‐[Hb+Mb]) was calculated. There was no significant interaction (measurement site × time) for intramuscular blood flow/deoxy‐[Hb+Mb] (proximal; 256 ± 138 a.u., middle; 384 ± 228 a.u., distal; 291 ± 197 a.u., *F* = 1.683, *p* = 0.076). These results suggest that the balance between oxygen supply and demand is uniformly regulated in the vastus lateralis along its length during intermittent submaximal isometric knee extension.

## 1 INTRODUCTION

Oxygen demand increases in active muscles during exercise, leading to an increase in blood supply to those muscles (Andersen & Saltin, 1985). If the oxygen supply does not match the oxygen demand, exercise performance becomes limited (Poole et al., 1988). Evaluating the dynamics and relationship between oxygen supply and demand in active skeletal muscles and clarifying how this balance changes over time would provide valuable insights into regional metabolism and circulatory regulation.

Near‐infrared spectroscopy (NIRS) is a powerful technique for assessing muscle deoxygenation (Hamaoka & McCully, 2019; McCully & Hamaoka, 2000). Studies have reported that muscle deoxygenation is heterogeneous along the length of individual muscles (Crenshaw et al., 2010; Kennedy et al., 2006; Kime et al., 2006; Miyamoto et al., 2013). For example, deoxygenation in the distal portion of the vastus lateralis is greater than in the proximal portion during knee extension (Crenshaw et al., 2010; Kennedy et al., 2006; Miyamoto et al., 2013) and cycling (Kime et al., 2006). This heterogeneity may be attributable to regional differences in intramuscular pressure, muscle activation patterns, and/or vessel structure (Kalliokoski, Scheede‐Bergdahl, et al., 2006; Quaresima et al., 2004). Moreover, it is speculated that blood flow within skeletal muscles (i.e., intramuscular blood flow), which is responsible for oxygen delivery, may contribute to regional differences in muscle deoxygenation (Andersen & Saltin, 1985). However, little is known about whether heterogeneity exists in intramuscular blood flow within individual muscles. This is likely due to the methodological limitations of intramuscular blood flow assessment during exercise.

Positron emission tomography (PET) has been used to measure regional blood flow (Heinonen et al., 2007; Kalliokoski et al., 2003, 2005). After injecting a radioisotope into the circulation, PET can be used to directly quantify blood flow in specific muscle regions based on the tracer concentration in the tissue (Heinonen, Kemppainen, Kaskinoro, Peltonen, Borra, Lindroos, Oikonen, Nuutila, Knuuti, Hellsten, et al., 2010). However, this method is limited by radiation exposure and has relatively low temporal resolution. To our knowledge, the only study to evaluate regional differences in blood flow along the entire length of the quadriceps femoris was conducted by Mizuno et al. (2003a). The authors reported that the increase in blood flow during exhausting cycling exercise was greater in the distal than in the proximal region. However, blood flow was assessed only twice (at baseline and 10 min after exercise). Therefore, changes in blood flow during exercise and immediately after exhaustion were not captured (Mizuno et al., 2003a). Another study demonstrated that vastus lateralis reoxygenation after fatiguing cycling exercise was delayed at the distal region (Kime et al., 2005). Therefore, the greater increase in distal intramuscular blood flow reported by Mizuno et al. (2003a) may reflect delayed recovery rather than a true exercise‐induced difference. Thus, whether regional differences exist in blood flow change patterns within the individual muscles of the quadriceps femoris during exercise remains unclear. To answer this question, a method capable of noninvasive, high‐temporal resolution measurements of intramuscular blood flow is needed.

In our previous study, we applied power Doppler ultrasonography, which has been used in clinical settings to assess organ and skeletal muscle perfusion (Izumi, Shiozawa, et al., 2025; Izumi, Yamamori, et al., 2025). Power Doppler ultrasonography is a noninvasive method for measuring blood flow in a specific region, primarily targeting superficial muscle layers (Dori et al., 2016; Heres et al., 2018; Joshua et al., 2006). This technique provides an estimate of the integrated Doppler power spectrum and detects frequency shifts caused by erythrocyte movement (Rubin et al., 1994). Thus, power Doppler ultrasonography enables noninvasive and continuous evaluation of intramuscular blood flow.

The present study aims to assess the regional differences in intramuscular blood flow, muscle deoxygenation, and their relationship during intermittent submaximal isometric knee extension. We hypothesized that intramuscular blood flow would be greater, and muscle deoxygenation more pronounced, in the distal than in the proximal region, leading to heterogeneity in the balance between oxygen supply and demand along the length of the vastus lateralis.

## 2 MATERIALS AND METHODS

### 2.1 Ethical approval

This study was approved by the Ethics Committee of the Research Center of Health, Physical Fitness & Sports at Nagoya University (Approval No. 21‐05) and was performed in accordance with the Declaration of Helsinki. Before the experiments, the purpose, risks, and benefits of the study were explained to the participants, and all participants provided written informed consent.

### 2.2 Experimental procedures

Thirteen healthy males participated in this study. All participants visited the laboratory on four separate days with an interval of at least 2 days. At the first visit, the participants practiced maximal voluntary contraction (MVC) and familiarised themselves with the intermittent isometric knee extension tasks. On the second to fourth visits, the participants underwent MVC measurements and completed the intermittent isometric knee extension protocol following warm‐up using submaximal contractions. To determine intra‐day and inter‐day reproducibility of measurements, four participants performed two bouts of exercise at least 15 min apart during a single visit, then revisited the laboratory several days later to perform the exercise again.

### 2.3 MVC

Isometric knee extension MVC measurement was conducted on the right leg using a custom‐designed dynamometer (M‐12297‐3; Takei Scientific Instruments Co. Ltd., Niigata, Japan) mounted on a force transducer (LTZ‐100KA; Kyowa Electronic Instruments Co. Ltd., Tokyo, Japan), as reported previously (Watanabe & Akima, 2009). During the knee extension tasks, the hip, chest, and ankle were tightly fixed to the seat using straps. The hip and knee joint were flexed at angles of 70° and 90°, respectively. The participants were instructed to cross their arms over their chest. Following submaximal contractions, the MVC test was performed three times with a rest of at least 2 min. The MVC test consisted of three phases: the rising phase (2–3 s), the sustained phase (≥3 s), and the relaxation phase (Watanabe & Akima, 2009). The supervisors encouraged the participants to apply their best effort. Knee extension force was recorded at 400 Hz using an analog‐to‐digital converter (PowerLab 16SP; ADInstruments, Melbourne, Australia) and saved on a personal computer (Mac Mini; Apple Inc., Cupertino, CA, US). The MVC force was determined as the highest value for each contraction. If the two greatest exerted forces between trials differed by >5%, an additional experiment was performed.

### 2.4 Intermittent isometric knee extension

The participants performed intermittent isometric knee extension exercises in the same posture as was used during the MVC measurements after resting for 10 min following MVC. Exercise was performed using a 50% contraction duty cycle (5‐s contraction, 5‐s relaxation) at a rate of 6 contractions/min. Eighteen contractions were performed at the force level of 50% MVC (Yoshiko et al., 2020). Real‐time force feedback was displayed on a computer monitor visible to the participants, and the target force was also displayed. Based on auditory feedback from the metronome, the participants performed muscle contractions and relaxations.

### 2.5 Intramuscular blood flow

**FIGURE 1:**
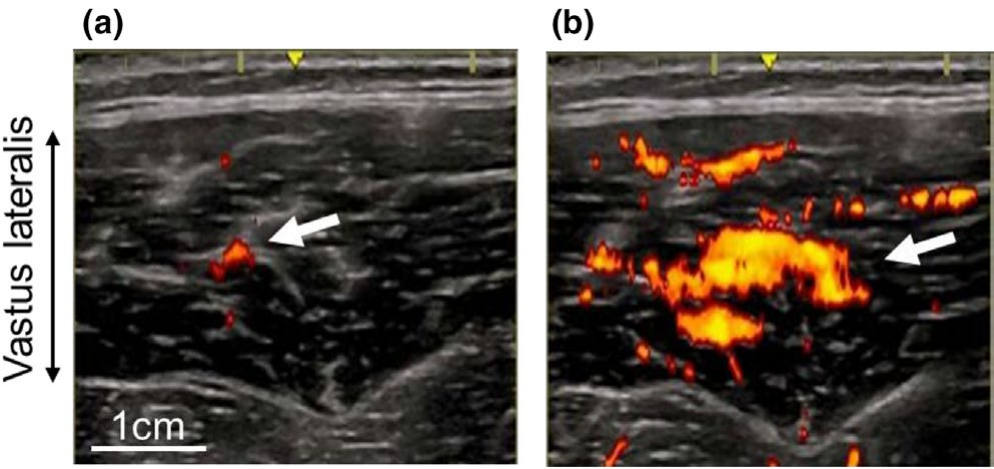
Representative power Doppler images within the middle portion of the vastus lateralis at baseline (a) and immediately after exercise (b). The arrows show power Doppler signals which indicate blood flow distribution.

As previously described (Izumi, Shiozawa, et al., 2025; Izumi, Yamamori, et al., 2025), intramuscular blood flow in the vastus lateralis during exercise was measured by power Doppler ultrasonography (LOGIQ e Premium; GE Healthcare, Wauwatosa, WI, US) equipped with a 12‐MHz linear‐array probe (probe width, 3.8 cm). The measurement sites were at the proximal third, middle, and distal third of the distance between the lateral epicondyle and the greater trochanter of the femur of the right thigh. The order of intramuscular blood flow measurements was randomized. To obtain transverse images, the probe was placed on the skin perpendicular to the predicted longitudinal axis of the vastus lateralis using a custom‐made styrene frame. Sufficient echo gel was applied to the probe to ensure acoustic contact without depression of the dermal surface. Care was taken to ensure that the probe positioning was kept constant during measurement. All ultrasonography system settings remained constant throughout power Doppler measurement, and the following acquisition parameters were used: frequency, 6.3 MHz; gain, 30.5 dB; depth, 7 cm (Izumi, Shiozawa, et al., 2025; Izumi, Yamamori, et al., 2025). The Doppler color area was configured to display the entire image field. Power Doppler images from the ultrasound monitor screen were captured using a capture device (DVI2USB 3.0; Epiphan Video, Ottawa, ON, Canada) and saved in AVI format at a sampling rate of 10 frames/s on a personal computer (ENVY; Hewlett‐Packard Japan, Tokyo, Japan). Representative images of power Doppler ultrasonography at the middle part of the vastus lateralis at baseline and immediately after exercise are shown in Figure 1a,b, respectively.

Intramuscular blood flow was analyzed using custom‐designed power Doppler signal‐measuring software, developed to calculate the pixel count of the power Doppler signal within the image (S‐22028 version 1.0.3; Takei Scientific Instruments Co. Ltd.) (Izumi, Shiozawa, et al., 2025; Izumi, Yamamori, et al., 2025). Intramuscular blood flow was determined by the relative area of the power Doppler signal in the region of interest using the following equation (Dori et al., 2016; Izumi, Shiozawa, et al., 2025; Izumi, Yamamori, et al., 2025): intramuscular blood flow (%) = (cross‐sectional area of power Doppler signal) ÷ (cross‐sectional area of region of interest) × 100.

Rectangular sections made at the frame where no muscle contraction was applied and where there was no movement artifact during muscle contraction were used to identify the region of interest in the vastus lateralis. As much muscle as possible was included in the selected area while avoiding visible fascia.

### 2.6 Muscle deoxygenation

As previously described (Akima & Ando, 2017), vastus lateralis deoxygenation was recorded using the NIRS system (Hb14; Astem Co. Ltd., Kanagawa, Japan) with a dual‐wavelength (770 and 830 nm) light‐emitting diode. The NIRS probe consisted of one light source and two photodiode detectors, with optode distances of 2 and 3 cm. Oxyhemoglobin+myoglobin, deoxyhemoglobin+myoglobin (deoxy‐[Hb+Mb]), and total hemoglobin+myoglobin were determined by measuring the light attenuation at wavelengths of 770 and 830 nm and analyzed using algorithms based on a modified Beer–Lambert law (Kime et al., 2010). The thickness of subcutaneous fat in the area where the NIRS signal was detected was measured using B‐mode ultrasonography (LOGIQ e Premium; GE Healthcare). By altering the scattering coefficient, subcutaneous fat thickness (i.e., path length) was used to minimize error and to determine the relative change in Hb+Mb and the absolute muscle oxygen saturation (StO_2_) by NIRS on a personal computer (ENVY; Hewlett‐Packard Japan) (Niwayama et al., 2007). The NIRS system provided the absolute StO_2_ values using the relative absorption coefficients derived from the light attenuation slope over a distance measured at two focal points from the light emission. The NIRS probe was attached perpendicular to the estimated longitudinal axis of the vastus lateralis at the distal neighbor to the ultrasound probe used to measure intramuscular blood flow. To prevent unwanted light interference, the probe was fixed using double‐sided adhesive tape and covered with elastic therapeutic tape. The NIRS signals were sampled at 2 Hz and transferred to a personal computer (ENVY; Hewlett‐Packard Japan) via a wireless connection (Bluetooth 2.0).

### 2.7 Data analysis

**FIGURE 2:**
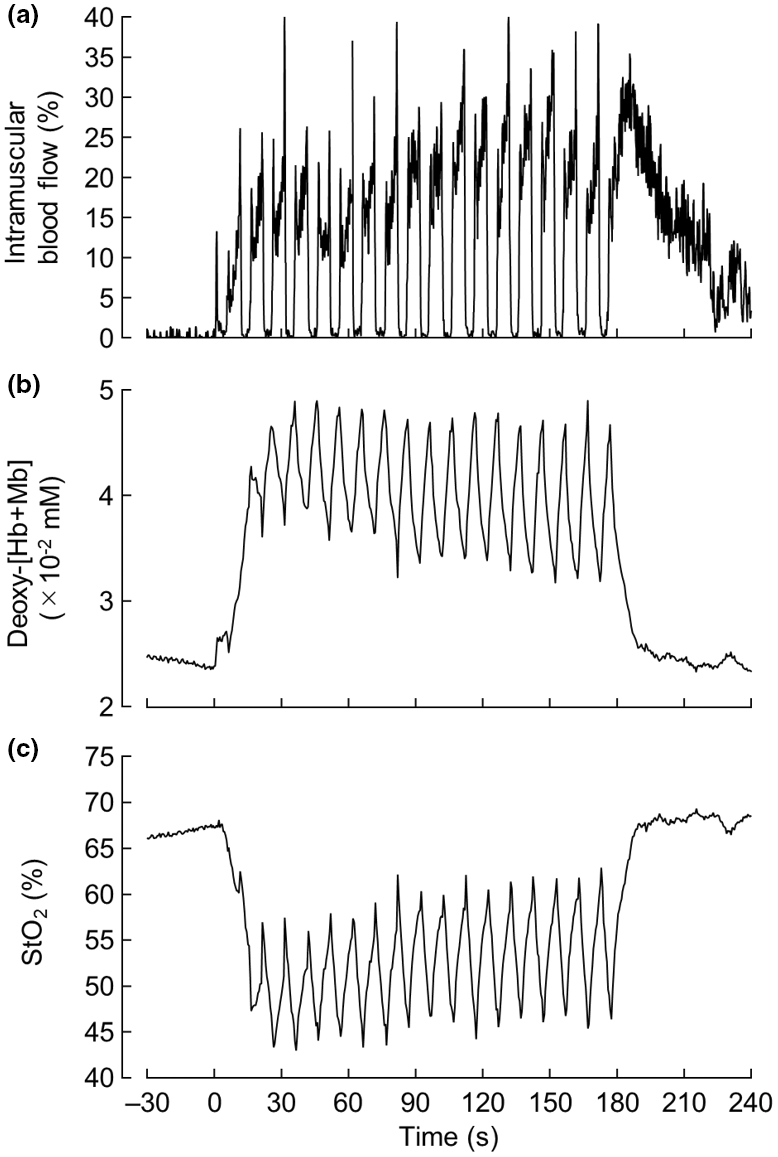
Representative recordings of intramuscular blood flow (a), deoxy‐[Hb+Mb] (b) and StO_2_ (c) at the middle portion of the vastus lateralis. Deoxy‐[Hb+Mb], deoxy‐hemoglobin+myoglobin; StO_2_, muscle oxygen saturation.

Exerted force was represented as the average of 18 contractions, excluding the first and last second of the 5‐s muscle contraction phase. Figure 2a–c shows representative recordings of intramuscular blood flow, deoxy‐[Hb+Mb], and StO_2_, respectively. The resting values of all parameters were averaged over 3 min, excluding the first and last minutes of the 5‐min baseline before exercise. Measurement variables were averaged during the relaxation phases, which were distinguished by feeding the power Doppler video frame by frame at each contraction and averaging every 30 s. Intramuscular blood flow and StO_2_ were represented as absolute changes from baseline (Δ). Intramuscular blood flow and deoxy‐[Hb+Mb] were normalized by the maximum. The ratio between intramuscular blood flow and deoxy‐[Hb+Mb] (intramuscular blood flow/deoxy‐[Hb+Mb]) was calculated as an index of the balance between oxygen supply and demand.

### 2.8 Kinetics analysis

The kinetics of Δintramuscular blood flow, Δdeoxy‐[Hb+Mb], and intramuscular blood flow/deoxy‐[Hb+Mb] were determined by fitting the data to a mono‐exponential model. Prior to fitting, the raw data were processed as follows. The baseline value was calculated as the mean of the data over 3 min, excluding the first and last minutes of the 5‐min baseline before exercise. The change in each variable from baseline (ΔY) was calculated for each average value during the relaxation phase. The kinetics of ΔY were modeled using the following mono‐exponential equation:

$$\mathrm{\Delta Y}\;\left(t\right)=A\cdot \left[1-e^{-\frac{t-\mathrm{TD}}{\tau }}\right]$$

where ΔY (*t*) represents the change in the variables at any given time (*t*); A is the plateau of the response above baseline; TD represents the time delay before the onset of the increase; and τ is the time constant. The TD and τ of the response were summed to obtain an estimate of the overall response of the parameters during exercise (mean response time [MRT]) (Koga et al., 2005, 2017). All curve fitting was performed using R‐studio (https://www.rstudio.com).

### 2.9 Statistical analysis

All values are reported as the mean ± standard deviation (SD). A two‐way analysis of variance with repeated measures (two‐way ANOVA with RM; measurement site × time) was used to identify differences in Δintramuscular blood flow, normalized intramuscular blood flow, normalized deoxy‐[Hb+Mb], ΔStO_2_, and intramuscular blood flow/deoxy‐[Hb+Mb]. One‐way ANOVA with RM was used to explore differences in the exerted force and the kinetics parameters. Kinetics parameter values exceeding 3 SDs from the mean or those identified by the Smirnov–Grubbs test were excluded. When ANOVA showed a significant difference, Bonferroni's post hoc test was performed. The coefficient of variation (CV) for each individual was calculated as the ratio of the SD to the mean. Between‐ and within‐day test–retest variability was assessed using the intraclass correlation coefficient (ICC) and classified according to Landis and Koch (1977) as 0.00–0.20 slight, 0.21–0.40 fair, 0.41–0.60 moderate, 0.61–0.80 substantial, and 0.81–1.0 almost perfect. Statistical analyses were performed using IBM SPSS Statistics software (version 27, IBM Corp., Tokyo, Japan). *p* < 0.05 was considered statistically significant.

## 3 RESULTS

**FIGURE 3:**
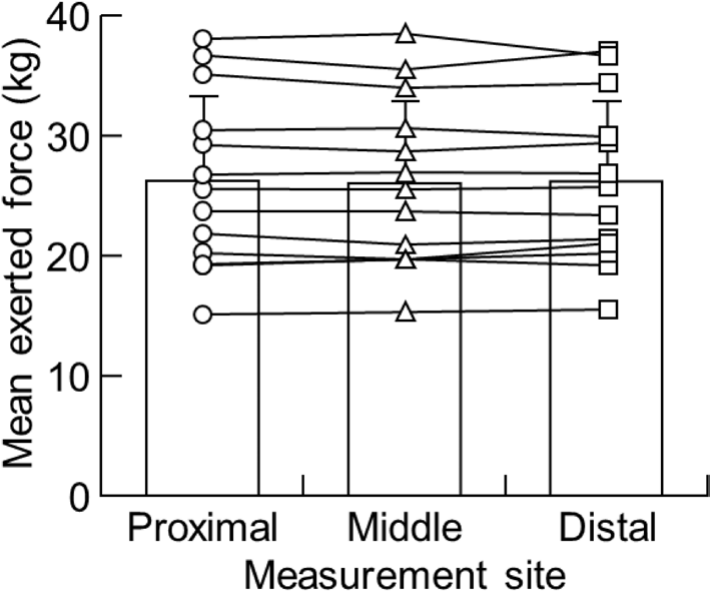
Mean exerted force during 18 isometric knee extensions across exercise.

The demographic and baseline characteristics of the participants were as follows: age, 19.4 ± 0.9 years; height, 170.3 ± 4.7 cm; weight, 54.4 ± 4.8 kg. There was no significant difference in the exerted force among three submaximal intermittent isometric knee extensions (*p* = 0.643) (Figure 3).

### 3.1 Intramuscular blood flow

**FIGURE 4:**
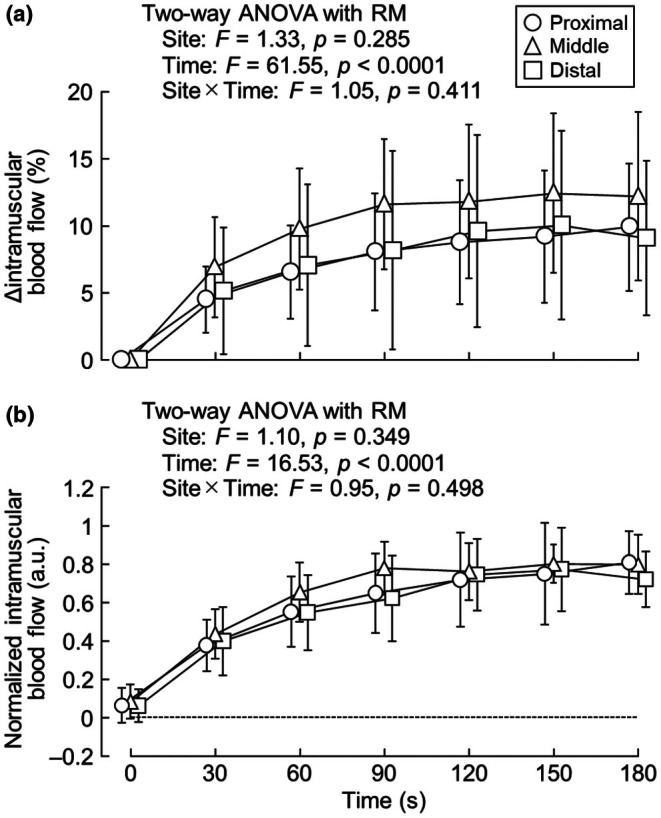
Δintramuscular blood flow (a) and normalized intramuscular blood flow (b) at different portions of the vastus lateralis. *n* = 13.

Figure 4a,b illustrates Δintramuscular blood flow and normalized intramuscular blood flow. There was no significant interaction between measurement site and time (*F* = 1.05, *p* = 0.411) and no main effect of measurement site (*F* = 1.33, *p* = 0.285). However, a main effect of time (*F* = 61.55, *p* < 0.0001) for Δintramuscular blood flow was observed, and Δintramuscular blood flow was significantly higher than at baseline at all subsequent time points (all *p* < 0.0001). Likewise, there was no significant interaction between measurement site and time (*F* = 0.95, *p* = 0.498) and no main effect of measurement site (*F* = 1.10, *p* = 0.349); however, a main effect of time (*F* = 16.53, *p* < 0.0001) was observed for normalized intramuscular blood flow. Normalized intramuscular blood flow significantly increased from baseline to 90 s (*p* = 0.021).

### 3.2 NIRS data

**FIGURE 5:**
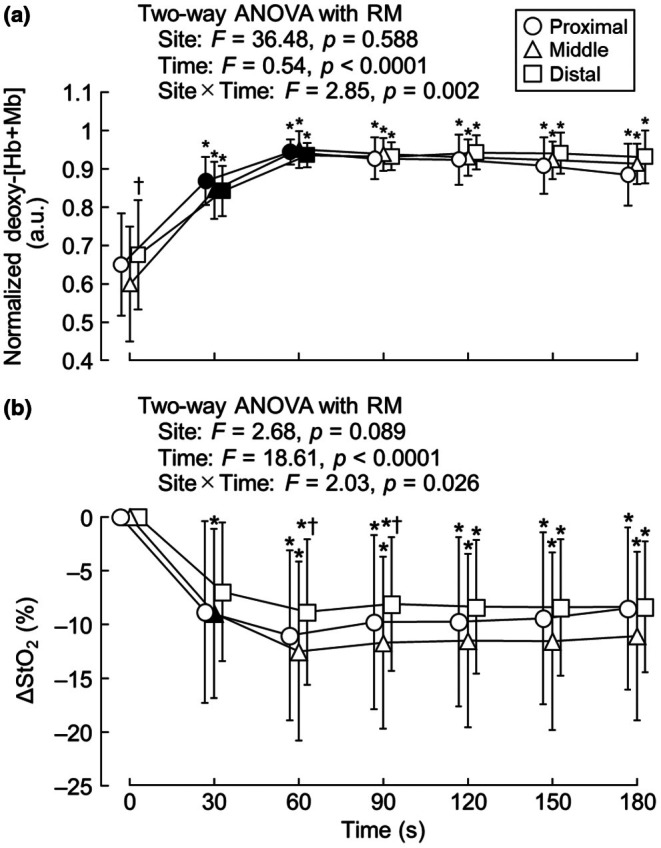
Normalized deoxy‐[Hb+Mb] (a) and ΔStO_2_ (b) at different portions of the vastus lateralis. Deoxy‐[Hb+Mb], deoxy‐hemoglobin+myoglobin; StO_2_, muscle oxygen saturation. *n* = 13. *p < 0.05 versus 0 s, † *p* < 0.05 versus middle. Solid symbols indicate data points that are significantly different from the one previous timepoint.

Figure 5a,b depicts normalized deoxy‐[Hb+Mb] and ΔStO_2_, respectively. There was a significant interaction between measurement site and time (*F* = 2.85, *p* = 0.002) and a main effect of time (*F* = 0.54, *p* < 0.0001), but there was no main effect of measurement site (*F* = 36.48, *p* = 0.588) for normalized deoxy‐[Hb+Mb]. Normalized deoxy‐[Hb+Mb] at all regions showed significantly higher values than at baseline at all timepoints (proximal, *p* = 0.003; middle, *p* = 0.001; distal, *p* = 0.015) and significantly increased at 60 s (proximal, *p* = 0.001; middle, *p* = 0.044; distal, *p* = 0.002). Compared with the middle portion, normalized deoxy‐[Hb+Mb] at the distal portion was significantly higher at baseline (*p* = 0.009).

For ΔStO_2_, there was a significant interaction between measurement site and time (*F* = 2.03, *p* = 0.026) and a main effect of time (*F* = 18.61, *p* < 0.0001), but there was no main effect of measurement site (*F* = 2.68, *p* = 0.089). Compared with baseline, ΔStO_2_ at the proximal and distal regions was significantly lower after 60 s (*p* = 0.044 and *p* = 0.014, respectively), and ΔStO_2_ at the middle portion was significantly lower at all timepoints (*p* = 0.040). Comparisons between the measurement sites showed that ΔStO_2_ at the distal portion was significantly higher at 60 and 90 s than at the middle portion (*p* = 0.004 and *p* = 0.006, respectively).

### 3.3 Intramuscular blood flow/deoxy‐[Hb + Mb]

**FIGURE 6:**
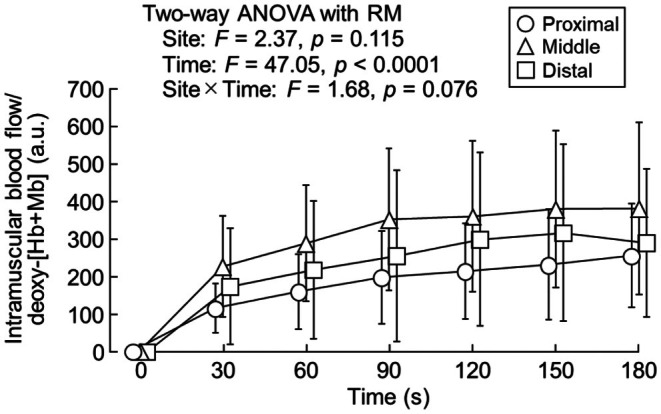
Changes in intramuscular blood flow/deoxy‐[Hb+Mb] at different portions of the vastus lateralis. Deoxy‐[Hb+Mb], deoxy‐hemoglobin+myoglobin. *n* = 13.

Figure 6 depicts the changes in intramuscular blood flow/deoxy‐[Hb+Mb]. There was no significant interaction between measurement site and time (*F* = 1.68, *p* = 0.076) and no main effect of measurement site (*F* = 2.37, *p* = 0.115), but time showed a main effect (*F* = 47.05, *p* < 0.0001) for intramuscular blood flow/deoxy‐[Hb+Mb]. Intramuscular blood flow/deoxy‐[Hb+Mb] significantly increased from baseline to 90 s (*p* = 0.013).

### 3.4 Responses kinetics

Table 1 shows the kinetics of Δintramuscular blood flow, normalized deoxy‐[Hb+Mb], and intramuscular blood flow/deoxy‐[Hb+Mb] following the onset of intermittent isometric knee extension. No significant differences were observed in any of the variables between the portions (*p* > 0.05).

**TABLE 1:** Kinetics of Δintramuscular blood flow, normalized deoxy‐[Hb+Mb], and intramuscular blood flow/deoxy‐[Hb+Mb] following onset of intermittent isometric knee extension.

	Plateau (%)	TD (s)	τ (s)	MRT (s)
Δintramuscular blood flow				
Proximal	11.13 ± 3.66	0.08 ± 0.18	35.19 ± 16.00	35.27 ± 15.99
Middle	12.43 ± 5.89	0.28 ± 0.35	28.44 ± 10.64	28.88 ± 10.48
Distal	8.51 ± 4.75	0.84 ± 1.57	43.57 ± 40.72	44.40 ± 41.44
Normalized deoxy‐[Hb+Mb]				
Proximal	0.92 ± 0.05	0.00 ± 0.00	6.03 ± 2.43	6.03 ± 2.43
Middle	0.94 ± 0.04	0.00 ± 0.00	7.49 ± 2.91	7.49 ± 2.91
Distal	0.94 ± 0.03	0.00 ± 0.00	7.14 ± 3.11	7.14 ± 3.11
Intramuscular blood flow/deoxy‐[Hb+Mb]				
Proximal	214.93 ± 64.41	0.00 ± 0.00	48.85 ± 45.57	48.86 ± 45.56
Middle	388.36 ± 214.51	0.05 ± 0.18	27.27 ± 12.98	27.43 ± 12.93
Distal	311.59 ± 215.98	0.23 ± 0.57	38.48 ± 36.94	39.19 ± 38.00

*Note*: Values are means ± SD.

Abbreviations: Deoxy‐[Hb+Mb], deoxy‐hemoglobin+myoglobin; MRT, mean response time; TD, time delay.

### 3.5 Reliability of intramuscular blood flow and StO_2_

Intramuscular blood flow at baseline (CV = 35.29 ± 26.85; ICC = 0.787) and during exercise (CV = 29.64 ± 10.38; ICC = 0.696) showed substantial between‐day reliability. Intramuscular blood flow at baseline (CV = 50.06 ± 6.28; ICC = 0.631) and during exercise (CV = 10.26 ± 2.70; ICC = 0.994) showed substantial to almost perfect within‐day reliability.

StO_2_ demonstrated almost perfect between‐day reliability at baseline (CV = 2.90 ± 1.16; ICC = 0.848) and during exercise (CV = 7.96 ± 5.64; ICC = 0.896). StO_2_ at baseline (CV = 1.18 ± 1.06; ICC = 0.571) and during exercise (CV = 2.91 ± 1.93; ICC = 0.818) showed moderate to almost perfect within‐day reliability.

## 4 DISCUSSION

We clarified the regional differences in intramuscular blood flow and muscle deoxygenation patterns of change, as well as their relationships, along the length of the vastus lateralis. Regional differences in intramuscular blood flow were not observed, but StO_2_ was significantly higher in the distal portion of the vastus lateralis than in the middle portion. In addition, intramuscular blood flow/deoxy‐[Hb+Mb] showed no significant regional difference within the vastus lateralis. These findings suggest that during intermittent submaximal isometric knee extension, oxygen supply is closely matched to oxygen demand, both temporally and spatially, in the vastus lateralis. The novel aspect of the present study is the simultaneous assessment of intramuscular blood flow and muscle deoxygenation at multiple regions along the length of the vastus lateralis using power Doppler ultrasonography and NIRS. To our knowledge, this is the first attempt to evaluate the dynamic balance between oxygen delivery and utilization within an individual muscle during exercise. This study provides valuable information regarding regional metabolism and peripheral circulatory regulation.

### 4.1 Regional differences in intramuscular blood flow

Although a previous NIRS study reported homogeneous deoxygenation across the length of the vastus lateralis (Koga et al., 2011), deoxygenation amplitudes alone do not fully characterize regional perfusion regulation. The temporal dynamics of intramuscular blood flow provide additional insight into the mechanisms of oxygen delivery and vascular reactivity during exercise. These features can vary independently of deoxygenation amplitude; therefore, assessment of intramuscular blood flow kinetics is necessary for understanding muscle oxygen transport dynamics. Contrary to our hypothesis, there were no regional differences in intramuscular blood flow changes along the length of the vastus lateralis during intermittent submaximal exercise (Figure 4a). To the best of our knowledge, Mizuno et al.'s study (2003a) is the only study to assess blood flow heterogeneity in the direction of the femoral long axis. Their study demonstrated that blood flow after exhaustive cycling exercise was reduced from the proximal to the distal region of the quadriceps muscle (Mizuno et al., 2003a), which is inconsistent with the results of the present study. In our study, intramuscular blood flow was measured continuously during exercise, whereas in the previous study, it was measured 10 min after exercise had ended (Mizuno et al., 2003a). In other words, the results obtained from the previous study may not reflect intramuscular blood flow during exercise; rather, they may reflect the slow recovery of intramuscular blood flow. Furthermore, as blood flow distribution is nonuniform among the quadriceps femoris (Kalliokoski et al., 2003, 2005; Rudroff et al., 2014), the results may differ depending on the muscles sampled. The discrepancy in the results between the present study and the previous study (Mizuno et al., 2003a) may be attributed to differences in the exercise protocol and/or modality. As intermittent submaximal isometric contractions were used to minimize task‐related variability in this study, intramuscular pressure elevation with the increase in exerted force augmented the regional metabolic requirements of the muscles. Therefore, if high‐intensity or exhaustive exercise is performed, regional differences in intramuscular blood flow may be observed, contrary to the results of this study. In addition, the difference in the exercise pattern may have contributed to differences in the results between the present study and the previous study (Mizuno et al., 2003a). During cycling exercise, both the hip and knee joints are used. The vastus muscles act only in knee extension, whereas the rectus femoris is a biarticular muscle involved in both knee extension and hip flexion. Thus, a paradox occurs with the rectus femoris because knee and hip joint motion occur simultaneously during pedaling (Andrews, 1987). Thus, the activation of each muscle is not uniform, and caution must be exercised when interpreting the results.

Several factors may influence the changes in intramuscular blood flow during exercise. First, the degree of muscle activation and intramuscular blood flow within the vastus lateralis are related to electromyographic activity during knee extension (Laaksonen et al., 2006). One study reported that the degree of muscle activation was uniform along the length of the vastus lateralis during knee extension (Wakahara et al., 2017). Although the degree of muscle activation was not assessed in the present study, it is likely that muscle activity would be consistent along the length of the vastus lateralis. Capillary density may also influence blood flow changes. Although capillary density depends on muscle fiber types (Lillioja et al., 1987), it has been reported that there are no regional differences in muscle fiber types along the length of the vastus lateralis (Lexell et al., 1983). As such, the finding of no regional differences in intramuscular blood flow in the present study may be explained by homogeneity in muscle activity or capillary density.

### 4.2 Regional differences in muscle deoxygenation

As oxygen diffuses from capillaries to myocytes longer blood transit time may enhance oxygen extraction (Kalliokoski et al., 2004). Blood transit time was significantly longer from the proximal to the distal portions of the quadriceps femoris during rest (Mizuno et al., 2003b). The present study showed that normalized deoxy‐[Hb+Mb] was significantly higher at the distal portion than at the middle portion at only baseline, which may be influenced by the slow blood transit time at the distal region.

Previous studies have shown regional differences in StO_2_ (Crenshaw et al., 2010; Esaki et al., 2005; Kennedy et al., 2006; Miyamoto et al., 2013; Quaresima et al., 2004). Among these studies, Crenshaw et al. (2010), Esaki et al. (2005), and Kennedy et al. (2006) reported that StO_2_ was significantly lower at the distal region of the vastus lateralis than at the proximal region during knee extension exercise. Similar results were reported by Miyamoto et al. (2013), who reported that StO_2_ was significantly lower at the distal region than at the middle region. Fascicle length shortening and contracting pennation angles result in a greater force component perpendicular to the aponeuroses, which in turn elevates intramuscular pressure (Kawakami et al., 1998). For example, in the gastrocnemius during plantar flexion, fascicle length shortening and pennation angle enlargement are greater in the distal portion than in the proximal portion (Miura et al., 2004). This suggests that intramuscular pressure would be greater at the distal portion than at the proximal portion. In addition, these changes in muscle architecture may cause regional blood flow disturbances in the muscle, which are related to the degree of muscle deoxygenation (Miura et al., 2001, 2004). Furthermore, a finite element model of skeletal muscle reported that intramuscular pressure was higher at the distal region than at the proximal and middle regions during muscle contraction (Wheatley et al., 2018). Taken together, it seems that muscle deoxygenation is enhanced at the distal portion of the muscle. Based on these previous studies, we hypothesized that StO_2_ would show lower values from the proximal to the distal region. However, there were regional differences in the pattern of StO_2_ changes, with StO_2_ being significantly higher in the distal region than in the middle region (Figure 5b), which was inconsistent with our hypothesis.

One explanation could be the influence of the depth at which the NIRS signal was detected. NIRS detects signals from tissues at depths of up to approximately half the distance between the light source and the detector (Chance et al., 1992). As the optode distance of the NIRS probe used in this study was 3.0 cm, the penetration depth into the tissue would have been approximately 1.5 cm. In other words, only the superficial portion could be measured in the middle part of the muscle where the muscle thickness is greatest. Meanwhile, the distal part where the muscle thickness is smaller could be measured in depth. Okushima et al. (2015) compared the deoxygenation kinetics between the superficial and deep portions of the rectus femoris and found that StO_2_ was higher in the deep portion than in the superficial portion. Moreover, the discrepancy in the results of StO_2_ between the present and previous studies (Crenshaw et al., 2010; Esaki et al., 2005; Kennedy et al., 2006; Miyamoto et al., 2013; Quaresima et al., 2004) may have been influenced by subcutaneous fat because it has been shown that subcutaneous fat thickness influences NIRS signals (Koga et al., 2011). In the studies by Crenshaw et al. (2010), Esaki et al. (2005), and Kennedy et al. (2006), there was no information about whether NIRS signals were corrected for subcutaneous fat thickness. Therefore, the NIRS data obtained in those studies may change if correction was performed. In addition, differences in the measurement sites may explain the discrepancies in the results between the present and previous studies. In this study, the NIRS probe was attached to the proximal, middle, and distal thirds of the vastus lateralis. In previous studies, the proximal third of the thigh length was used as the measurement site for the proximal region, with the measurement site being 8–10 cm distal to the proximal probe (Crenshaw et al., 2010). Kennedy et al. (2006) measured StO_2_ at the distal third of the thigh length, with the proximal being 10 cm proximal to the distal probe. Thus, differences in the detection depth of NIRS signals and the attachment position of the NIRS probes may explain the discrepancies in the results between the present and previous studies.

### 4.3 Regional differences in intramuscular blood flow/deoxy‐[Hb+Mb]

The changes in intramuscular blood flow/deoxy‐[Hb+Mb] were not significantly different among the different regions of the vastus lateralis (Figure 6). This ratio is an index that comprehensively evaluates the balance between oxygen supply and demand, which cannot be clarified by measuring oxygen supply or utilization separately. The lack of regional differences in the ratio may indicate that local vascular regulation mechanisms ensure the matching of oxygen supply and demand uniformly along the vastus lateralis. Local vasoactive substances, such as nitric oxide, prostaglandins, and adenosine, regulate microvascular blood flow, functioning to adjust regional oxygen delivery to meet oxygen demand (Heinonen, Kemppainen, Kaskinoro, Peltonen, Borra, Lindroos, Oikonen, Nuutila, Knuuti, Boushel, & Kalliokoski, 2010; Kalliokoski, Langberg, et al., 2006). Thus, although StO_2_ alone showed regional differences, the combined index (i.e., intramuscular blood flow/deoxy‐[Hb+Mb]) suggests that oxygen supply–demand matching is tightly regulated across the length of the vastus lateralis.

### 4.4 Regional differences in the kinetics profiles of intramuscular blood flow and muscle deoxygenation

There were no significant regional differences in the MRT of Δintramuscular blood flow, normalized deoxy‐[Hb+Mb] and intramuscular blood flow/deoxy‐[Hb+Mb] within the vastus lateralis (Table 1). MRT represents the overall response of the parameters during exercise (Koga et al., 2005, 2017). Koga et al. (2011) reported that the kinetics profiles of muscle deoxygenation were not different between the distal and proximal portions of the vastus lateralis, which is consistent with the present study. To our knowledge, no studies have compared the kinetics of intramuscular blood flow or intramuscular blood flow/deoxy‐[Hb+Mb] within an individual muscle. Oxygen supply and demand could be regulated by muscle fiber types (Behnke et al., 2003; Ferreira et al., 2006). The proportions of type I and type II fibers are not heterogeneous along the length of the vastus lateralis (Lexell et al., 1983). The lack of regional differences is thought to be related to the relatively uniform spatial distribution of muscle fiber types.

In general, intramuscular blood flow and muscle deoxygenation have been assessed at the middle portion of muscles (Akima & Ando, 2017; Heres et al., 2018; Izumi, Yamamori, et al., 2025). Non‐heterogeneity in these parameters within individual muscles may indicate that measurements at the middle portion are representative of the entire vastus lateralis muscle.

### 4.5 Experimental limitations

This study had several limitations. First, the SD values of each parameter were large in the kinetics analysis (Table 1). Therefore, the conclusion of no regional differences should be interpreted cautiously, considering this high variability. Second, it is necessary to consider the possibility that the homogeneity in oxygen demand–supply matching observed in this study is specific to the vastus lateralis during moderate‐intensity intermittent isometric exercise. The results may differ if we studied other muscles, higher‐intensity exercise, or dynamic exercise. For example, a previous study reported that neuromuscular activation of the rectus femoris increased across the entire muscle during knee extension, whereas only the proximal region showed increased activation during hip flexion (Watanabe et al., 2012). This regional difference in neuromuscular activation may influence hemodynamics and muscle deoxygenation. As the muscle fiber types being recruited and the status of the energy supply are closely related, it has been proposed that oxygen availability regulates the recruitment of type II fibers (Pringle et al., 2003). Additionally, muscle blood flow increases more during dynamic exercise than during isometric exercise because of the muscle pump function caused by changes in muscle fiber length (Laaksonen et al., 2003). Cardiovascular parameters, such as muscle oxygenation and blood pressure, are also affected by the mode of exercise (Kounoupis et al., 2022; Lee et al., 2021). Third, we recruited only young men in this study. Hemodynamics have been reported to vary depending on sex, age, and disease states. For instance, muscle deoxygenation during intermittent (Ansdell et al., 2019) and sustained (Lee et al., 2021) isometric knee extension is significantly greater in young men than in young women. Moreover, the femoral artery hyperemic response induced by dynamic knee extension is significantly greater in young women than in young men (Parker et al., 2007). In addition, studies have demonstrated that exercise tolerance declines with aging (Dorff et al., 2024) and disease (Askew et al., 2005; Senefeld et al., 2019). In such participants, uneven oxygen supply and demand as a result of decreased vascular reactivity may be a factor limiting exercise tolerance. Future studies involving a broader participant demographic are needed to examine differences in the responses to exercise. Finally, the measurement regions for intramuscular blood flow and muscle deoxygenation were not the same. Muscle deoxygenation was assessed inside the superficial muscle, while intramuscular blood flow was examined to the depth of the vastus lateralis.

## 5 CONCLUSION

We investigated regional differences in the intramuscular blood flow and muscle deoxygenation patterns of change, as well as their relationship, in the vastus lateralis during intermittent submaximal isometric contractions. Clarifying these differences may be beneficial to elucidate the mechanisms underlying regional vascular and metabolic regulation in skeletal muscle during exercise. Although no regional differences were observed in intramuscular blood flow, StO_2_ was significantly lower in the middle than in the distal portion of the vastus lateralis. Additionally, no significant regional differences were detected in the patterns of change in intramuscular blood flow/deoxy‐[Hb+Mb] along the length of the vastus lateralis. These findings suggest that the vastus lateralis exhibits regulatory mechanisms that maintain a homogeneous balance between oxygen supply and demand along its length during intermittent submaximal isometric knee extension.

## AUTHOR CONTRIBUTIONS

Conception of design of the work: K.I., K.K., N.T., and H.A. Acquisition, analysis, or interpretation of the data for the work: K.I., K.K., Y.K., N.T., and H.A. Drafting of the work or revising it critically for important intellectual content: K.I., K.K., Y.K., N.T., and H.A. All authors approved the final version of the manuscript and agree to be accountable for all aspects of the work in ensuring that questions related to the accuracy or integrity of any part of the work are appropriately investigated and resolved. All persons designated as authors qualify for authorship, and all those who qualify for authorship are listed.

## FUNDING INFORMATION

Grant‐in‐Aid for Challenging Research Exploratory from the Ministry of Education, Culture, Sports, Science and Technology Grants (22K19728) and JST SPRING grant (JPMJSP2125).

## CONFLICT OF INTEREST STATEMENT

None declared.

## Data Availability

The datasets generated and analyzed during the current study are available from the corresponding authors upon reasonable request.
